# Dysmenorrhea Symptoms Interference Scale (DSI) in Serbian - Translation and psychometric properties evaluation

**DOI:** 10.1371/journal.pone.0353688

**Published:** 2026-07-16

**Authors:** Dejan Mihajlovic, Momir Dunjic, Nenad Sulovic, Leonida Vitkovic, Kristina Brajovic Car, Slavica Aksam, Dusica Kocijancic Belovic, Jelena Dotlic

**Affiliations:** 1 Department for Obstetrics and Gynecology, Clinical Health Center Kosovska Mitrovica, Kosovska Mitrovica, Kosovo, Serbia; 2 Faculty of Medicine, University of Pristina temporarily settled in Kosovska Mitrovica, Kosovska Mitrovica, Kosovo, Serbia; 3 Institute for Histology and Embryology, Faculty of Medicine, University of Pristina temporarily settled in Kosovska Mitrovica, Kosovska Mitrovica, Kosovo, Serbia; 4 The Faculty of Media and Communications, University Singidunum, Belgrade, Serbia; 5 Clinic of Obstetrics and Gynecology, University Clinical Center of Serbia, Dr Koste Todorovica 26, Belgrade, Serbia; 6 Medical Faculty University of Belgrade, Dr Subotica 8, Belgrade, Serbia; Kindai University: Kinki Daigaku, JAPAN

## Abstract

**Background and Objective:**

Dysmenorrhea, painful menstruation, affects 20–90% of women and it can significantly impair their quality of life. To evaluate the severity and consequences of dysmenorrhea specific questionnaires have been developed. The aim of this study was to translate and culturally adapt the Dysmenorrhea Symptoms Interference Scale (DSI) for the Serbian-speaking population and assess its psychometric properties.

**Materials and Methods:**

This study included 350 Serbian-speaking female students with primary dysmenorrhea symptoms at least once during the past 12 months attending health sciences at the University of Pristina temporarily settled in Kosovska Mitrovica. They completed the general socio-epidemiologic questionnaire, the DSI and Depression, Anxiety and Stress Scale-21 (DASS-21). The DSI was translated according to the recommended methodology for the cultural adaptation of questionnaires, and its psychometric characteristics (internal consistency, validity based on internal structure, discriminant and convergent validity) were tested.

**Results:**

There were no major changes in the items during the process of translation or validation. The Cronbach’s alpha coefficient for the whole scale was 0.928, whereas if an item was deleted, it was above 0.8 for all items. The McDonald’s omega coefficient was 0.951, indicating good internal consistency. The CI‒TC coefficients were greater than 0.40 for all the items, indicating that all the items were significant elements of the DSI. Exploratory factor analysis confirmed one-factor structure of the Serbian DSI version (total variance explained 64.16%). Confirmatory factor analysis showed adequate values of fit indices (goodness of fit 0.998; root mean square error of approximation 0.032). The DSI significantly correlated (ρ = 0.520; p = 0.001) with DASS-21, confirming its adequate convergent validity. Moreover, DSI scores significantly (p = 0.001) differed between students with mild and severe dysmenorrhea proving good discriminant validity.

**Conclusions:**

The Serbian version of the DSI showed adequate psychometric properties and it can be applied both in research and in broad clinical use.

## Introduction

Dysmenorrhea is defined as painful menstruation. The typically cramping pain may radiate to the thighs or lower back and be accompanied by nausea and vomiting, headache, diarrhea, fatigue, dizziness, and breast tenderness. The pain starts with the onset of menstruation and usually resolves within two or three days. Dysmenorrhea mostly occurs in adolescents (12–24 years) and young adults (up to 29 years of age) [1–3]. It affects 20–90% of women during the reproductive age, but regional and national estimates are lacking due to differences in the definitions and dysmenorrhea severity levels in previous investigations. Prevalence of dysmenorrhea in European ranges from 45–97% in general population and from 41–62% among hospitalized patients. In studies that included women with occasional and light symptoms rate of dysmenorrhea was over 80%. On the other hand, 10–15% of women have severe dysmenorrhea each month which can disturb normal daily activities at work, home, or school [4–6].

Risk factors for dysmenorrhea include smoking, high or low body mass index, younger age at menarche, longer menstrual cycles, longer and heavier menstrual flow, nulliparity and high level of stress [7,8].

If there is no underlying pelvic pathology or anatomic variant that might be the cause of pain the dysmenorrhea is classified as primary. Contrary, secondary dysmenorrhea is associated with different gynecological conditions out of which the most common causes of painful menstruation are endometriosis, adenomyosis or myoma. The adequate early diagnosis and distinction between primary and secondary dysmenorrhea is important for choosing optimal therapy and predicting potential complications [9–11].

The pathophysiology of primary dysmenorrhea is still under investigation, but it is mostly considered to be caused by rise in prostaglandin levels which lead to increased uterine pressure and contractions. Impaired uterine perfusion, ischemia, hypoxia due to contractions of higher intensity and metabolites from anaerobic metabolism also contribute to dysmenorrheal [2,12].

Severe dysmenorrhea can significantly impair women’s daily activities, sleep, concentration and productivity, relationships with family and friends, as well as the overall feeling of quality of life [3,4]. Studies reported that around 40% of women carried out fewer activities, 50% avoid exercising while some were even unable to do anything during painful menstruations. Sleep quality worsens during menstruation compared to other menstrual cycle phases. Presence of the pain significantly impacts academic performance (primary and secondary school as well as university education). Therefore, dysmenorrhea is usually associated with increased levels of anxiety and depression [5,6,13–15].

To evaluate the severity of dysmenorrhea and the effects of its treatment validated outcome measures are needed. Although there are quite a few instruments for investigating pain levels, better data can be obtained by instruments specifically designed for dysmenorrhea patients that enable multidimensional assessment all of the symptoms and its interference with quality of life and daily activities [16]. Therefore, the Dysmenorrhea Symptoms Interference Scale (DSI) was developed. Currently, there are available translations of DSI into several languages, but few of them were adequately validated. Moreover, no translation for the Serbian language exists [17,18].

The Serbian southern province of Kosovo has had numerous socioeconomic, educational and health challenges as a result of previous armed conflict between Serbian (mostly living in North Kosovo) and Albanian communities (generally living in the remainder of Kosovo). Factors such as limited business opportunities and high rates of unemployment, ongoing ethnic tensions, migrations, the availability of inexpensive illicit substances and alcohol as well as the presence of foreign military and nongovernmental organizations increase the level of overall stress among the population [19]. Thus, it could be anticipated that different psychosomatic illnesses are prevalent in the population of Kosovo, including dysmenorrhea in young women, who are trying to obtain the best possible education in such difficult circumstances [20].

Therefore, the study aim was to translate, culturally adapt and validate the Dysmenorrhea Symptoms Interference Scale (DSI) in the Serbian language, which could be used in the everyday counseling of women with painful menstruations both in Kosovo and central Serbia.

## Materials and methods

This prospective study was conducted at the University of Pristina, temporarily seated in Kosovska Mitrovica, over a two-month period (April 15th to June 15th, 2023). Female students from all school years attending health sciences (studies of medicine, dentistry and nursing) were included in the study. As dysmenorrhea is most common among women aged 15–25 years students were chosen for the study as the common proxy for young and healthy populations [5,6]. Before the study commenced, the sample size was calculated based on the number of female students attending the University (N = 323 respondents, confidence interval 95%, margin of error 5%, study power 95.3%).

The study inclusion criteria were as follows: aged ≥18 years, complaining on dysmenorrhea at least once during the past 12 months, having primary dysmenorrhea and being a health sciences student fluent in the Serbian language. The study exclusion criteria were as follows: verified gynecological and/or psychiatric diseases, which, according to the literature, may be the cause of secondary dysmenorrhea (myoma, adenomyosis, endometriosis, anxiety, depression, etc.), declining participation or fulfilling less than 90% of the questionnaire.

The recruitment of respondents was carried out on weekdays during regular compulsory theoretical and practical classes and in agreement with lecturers who were all very helpful and willing to allow the survey. Before being included in the study, student was thoroughly informed by the researchers about the signs and symptoms as well as the causes of dysmenorrhea and the purpose of the study. All students gave their signed consent for participation after which they were offered to fill-in a set of questionnaires in the paper form. Students filled-in the questionnaires alone at their seats which took up to 20 minutes. Researchers were present for all questions. The response rate was 83.3%. Students who declined participation had no specific differences from the others, but were generally not interested in taking surveys or felt tired and opted to make a 15 minutes break from lectures. Finally, we had to exclude 6 students as they filled in less than 90% of the questionnaire and 29 students who reported having conditions that might cause secondary dysmenorrhea. The study was approved by the Ethics Committee of the Faculty of Medicine, University of Pristina, temporarily settled in Kosovska Mitrovica (05.04.2023. Approval 09–799).

### Instruments

The DSI is a 9-item scale that measures how dysmenorrheal symptom affects physical, mental and social activities of a woman. The items are scored from 1 (not at all) to 5 (very much). The scores given to the items are summed up and the final scale score is calculated as the mean of item scores (range 1–5). The higher the final score of the scale, the greater the impact of dysmenorrhea on the mentioned daily activities. The DSI was developed as a one-factor construct in English language and validated in the United States for use among women aged 14–42 years. It can be administered in paper or electronic form. Self-administration is preferable, but it can also be used in interviews. Original testing and previous validations showed very good psychometric properties of DSI making it useful for both clinical work and research [17,18].

The Depression, Anxiety and Stress Scale – 21 items (DASS 21) is one of the most reliable and commonly used instruments designed to assess the severity of general psychological distress and assess negative emotional states in clinical and non-clinical settings. It is a self-reported questionnaire that comprises 21 items/statements divided into three domains (depression, anxiety and stress) each with 7 items/statements. The statements refer to the period of the previous week. Respondents rate each statement from 0 (not at all) to 3 (mostly or almost always). The cut-off values based on the original validation study, and recommended in the scoring instructions, for the severity of the mental condition are: ≥ 10 for the depression scale, ≥ 8 for the anxiety scale and ≥15 for the stress scale. Finally, the original scores are multiplied by two to obtain a total DASS 21 score. Higher scores indicate a more severe disorder [21,22].

### DSI translation and content validity

The license to use the DSI questionnaire was obtained from ePROVIDE™, Mapi Research Trust, Lyon, France (No 2211755, 18.08.2022.) [18]. The DSI was translated according to the internationally accepted methodology to generate a version that was semantically and conceptually as close as possible to the original [23]. Translation of the original English DSI to Serbian language (“forward translation”) was performed by two independent translators (study authors) native Serbian speakers fluent in English language. The “backward translation” (from Serbian back to English) was completed by the third translator (native English speaker fluent in Serbian language) who was blinded to the original questionnaire. Finally, translators discussed all items to generate a version which would be the most appropriate for the cultural environment of Serbia. The final Serbian version of the DSI was tested on 10 female students to check if any of the items were incomprehensible, confusing or culturally inappropriate. As there were no remarks on clarity and understanding of items and as there were no suggestions to include additional items, the final version was generated and applied in this study.

### Statistical analysis

Methods of descriptive and analytical statistics (number, percent, mean value, standard deviation – SD and χ2 test) were used to briefly illustrate the study population. As this study is a part of a larger investigation, description of the study sample was previously performed in SPSS (version 21 IBM, USA) program, while all validity analyses for this study were performed in JASP (version 0.19.3 University of Amsterdam) statistical software.

To describe DSI questionnaire, we analyzed minimal and maximal values, mean and standard deviation. Data distribution was investigated by Kolmogorov-Smirnov test (p > 0.05 indicates normal distribution) and skewness (if data do not weight toward score extremities skewness value should between −1 and 1). Moreover, Mardia’s test was also done to assess multivariate normality [23].

### Validity evidence based on internal structure

To examine the validity evidence based on internal structure of the Serbian DSI version we performed exploratory factor analysis (EFA) and the confirmatory factor analysis (CFA). For these analyses the sample was divided in two equal (n = 175) random groups (one as EFA sample and one as CFA sample) as suggested for confirmation of EFA findings by CFA on a separate sample and to avoid testing the data against itself [23,24]. For obtaining reliable results of questionnaire validation it is commonly recommended to include 5–10 participants per item [24]. As the DSI had 9 items the sample size of 175 students per group was considered as adequate. The Kaiser-Meyer-Olkin (KMO) test and Bartlett’s test of sphericity were analyzed to assess the adequacy of sampling (KMO values above 0.7) and to determine if the correlations between variables are significant enough for factor analysis (significant p-value in Bartlett’s test) [23,24].

In EFA minimum residuals factoring method was applied to extract the factors. Factor loadings (correlations between the questionnaire items and extracted factors) were analyzed using the cut-off points of 0.40. If eigenvalues of the factor are above 1.0 the factor is significant. Additionally, parallel analysis was performed to ensure that the selection of latent factors is properly conducted. Finally, communality indices (CI – the sum of squared factor loadings) of questionnaire items were evaluated. Item CI should be ≥ 0.4 for the item to be kept as the part of the questionnaire [23,24].

In the CFA, as the model goodness-of-fit estimators we assessed goodness of fit index (GFI), comparative fit index (CFI), Tucker-Lewis Index (TLI), normative fit index (NFI), Non-Normed Fit Index (NNFI), Parsimony Normed Fit Index (PNFI), McDonald fit index (MFI), Relative Fit Index (RFI), root mean square error of approximation (RMSEA) and SRMR (Standardized Root Mean Square Residual). The Unweighted Least Squares (ULS) estimator was chosen for the analysis as it optimally corresponded with our non-normally distributed data in a limited sample. For the model to be adequate values of RMSEA and SRMR should be ≤ 0.08 (with upper limit of the confidence interval below 0.1) while other indices should optimally be ≥ 0.90. Higher values of PNFI indicate better model fit [23,24].

### Internal consistency

Internal consistency of the Serbian DSI version was evaluated by Cronbach’s alpha and McDonalds Omega coefficients. Alpha coefficient is based on the average inter-item correlation and relies on strict assumptions that all items load equally on the underlying factor. Omega coefficient is based on a factor analysis model. It accounts better for multidimensionality and is therefore considered to be more accurate. For both coefficients satisfactory values are above 0.7 [23]. Hotelling T–square test (HT2) shows whether or not there is a significant difference between obtained mean score values of all DSI items together and the hypothetic case in which items have equal scores [23].

Discriminating characteristics of the questionnaire items were tested by Corrected Item – Total Correlation (CI–TC) analysis. This analysis showed the relationships of one item with the score of remaining questionnaire items. An item is considered as adequate part of the questionnaire if CI–TC is ≥ 0.40 [23].

### Convergent and discriminant validity

The convergent validity was investigated by Spearman’s correlation coefficient. Various literature data demonstrated the significant association of dysmenorrhea, stress and depression [15,20]. Therefore, the final DSI score was correlated with the scores of DASS 21. DASS 21 is established and widely used questionnaires for evaluation of different psychological disturbances that can occur during menstrual cycle of some women [22,25]. Finally, discriminant analysis was assessed by comparing the DSI scores between students with mild and severe dysmenorrhea to establish the potential of DSI to differentiate between patient groups [23]. Mild dysmenorrhea was considered if the pain was reported to be manageable and not lasting longer than the first 2–3 days of the cycle. Patients have severe dysmenorrhea if the intense and prolonged pain is followed by systemic symptoms causing interference with daily activities [1,3].

## Results

### Description of the study sample

The study incorporated 350 female students who in average had 20.58 + /- 1.97 years (range 18–25 years). Majority of students were studying medicine (82.9%). Dentistry was attended by 9.4% and nursing 7.7% of students. They were generally healthy (14.6% had chronic and 12.9% had gynecological illnesses), with regular menstrual cycles (89.1%), but no previous pregnancies (77.4%). Few investigated students used hormone contraception (3.4% only as contraception and 8% to treat menstrual cycle irregularities, premenstrual syndrome and/or dysmenorrhea).

Investigated students mostly reported that dysmenorrhea symptoms were present since the time of menarche (66.0%) during almost all cycles (42%) and for the first two days of menstruation (77.7%). Majority of students stated that the pain was intensive but bearable (36.6%) although 57.1% of students regularly took analgesics for the pain.

### DSI items and scores

The simple literal translation was adequate for almost all DSI items and just minor changes were made during the process of translation and validation. Average DSI item scores are presented in Table 1. Data were not normally distributed by standard means of assessment as well as (p = 0.001) multivariate normality testing (Mardia’s skewness and kurtosis p = 0.001). Still, thorough evaluation of skewness per item showed that it was appropriate for all items.

**Table 1:** Description of the Serbian DSI.

Items	Minimum	Maximum	Mean	Standard Deviation	Skewness
**Physical activities**	1.00	5.00	2.46	1.166	0.504
**Sleep**	1.00	5.00	2.16	1.160	0.664
**Daily activities**	1.00	5.00	2.36	1.09	0.502
**Work**	1.00	5.00	2.31	1.15	0.525
**Concentration**	1.00	5.00	2.48	1.23	0.383
**Enjoyment of life**	1.00	5.00	2.45	1.29	0.505
**Leisure activities**	1.00	5.00	2.37	1.26	0.553
**Social activities**	1.00	5.00	2.13	1.19	0.788
**Mood**	1.00	5.00	3.27	1.32	0.161
**DSI score**	1.00	5.00	2.44	0.96	0.418

There were no significant differences in the frequency of grades per item (1–5) in our sample. All of the items were graded with marks from 1 to 5 meaning that at least some of the investigated female students had all of the assessed complaints. Still, only 7.5% of students had a high final DSI score (above four). The highest average score was achieved for item #9 (mood), while items #2 and #8 (sleep and social activities) had the lowest average scores.

### Validity evidence based on internal structure

**Fig 1:**
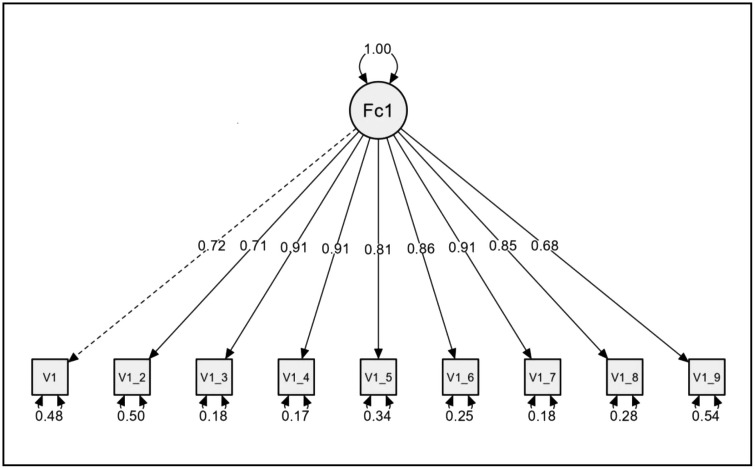
Confirmatory factor analysis of Serbian DSI (standardized factor loadings).

As sampling was adequate (KMO = 0.885; Bartlet p = 0.001) factor analysis was performed. On EFA we obtained one factor for the Serbian DSI version just like in the original validation (Table 2). The factor loadings and communality indices for all the items were >0.4. Total variance explained by the extracted factor was 64.16%. Parallel analysis confirmed that only one factor should be retained (Table 3). Finally, CFA (Fig 1) showed that for the obtained model (χ^2^ = 234.617; df = 27; p = 0.001) all fit indices were appropriate (Table 4).

**Table 2:** Serbian DSI internal consistency and exploratory factor analysis statistical parameters.

Items	Cronbach’s Alpha if item deleted	Corrected Item-Total Correlation	Factor loadings	Communalities
Physical activities	0.924	0.648	0.726	0.526
Sleep	0.926	0.626	0.702	0.492
Daily activities	0.914	0.823	0.872	0.760
Work	0.914	0.820	0.872	0.760
Concentration	0.918	0.751	0.807	0.651
Enjoyment of life	0.916	0.776	0.828	0.686
Leisure activities	0.913	0.826	0.873	0.763
Social activities	0.917	0.773	0.828	0.685
Mood	0.929	0.600	0.671	0.451

**Table 3:** Parallel analysis.

Factors (* indicates which factor should be retained)	Real data component eigenvalues	Simulated data mean eigenvalues
Factor 1*	5.762	1.252
Factor 2	0.798	1.166
Factor 3	0.587	1.097
Factor 4	0.457	1.043
Factor 5	0.424	0.994
Factor 6	0.338	0.947
Factor 7	0.302	0.891
Factor 8	0.189	0.847
Factor 9	0.145	0.762

**Table 4:** Confirmatory factor analysis model fit measures.

Fit measures	Value
Goodness of fit index (GFI)	0.998
Comparative Fit Index (CFI)	0.998
Tucker-Lewis Index (TLI)	0.998
Root mean square error of approximation (RMSEA)	0.032
RMSEA 90% confidence interval lower bound	0.001
RMSEA 90% confidence interval upper bound	0.056
Standardized root mean square residual (SRMR)	0.048

### Internal consistency

Cronbach’s alpha coefficient of the Serbian DSI version was 0.928 while McDonald’s omega coefficient was 0.951 indicating good internal consistency. Value of Cronbach’s alpha coefficient if item deleted was above 0.80 for all items (Table 2). If the item #9 (mood) was deleted Cronbach’s alpha coefficient was the highest (coefficient = 0.929) while if item #7 (leisure activities) was deleted it was the lowest (coefficient = 0.913).

Based on Hotelling’s T-Squared test, there was a significant difference between item scores (HT^2^ = 345.803; F = 42.358; p = 0.001). The values of the CI-TC coefficients for the DSI in Serbian population were higher than 0.40 for all items, with the lowest of 0.601 for item #9 (mood). Consequently, all of the items were significant elements of the questionnaire.

### Convergent and discriminatory validity

The Serbian version of DSI displayed an adequate convergent validity as its total score significantly correlated with DASS 21 total score (ρ = 0.520; p = 0.001). Moreover, DSI total score positively correlated with Depression (ρ = 0.453; p = 0.001), Anxiety (ρ = 0.441; p = 0.001) and Stress (ρ = 0.449; p = 0.001) domain scores of DASS 21.

Significant differences were registered regarding DSI scores between students with mild and severe dysmenorrhea symptoms (F = 29.391; p = 0.001) which indicated that DSI can adequately differentiate patient groups.

## Discussion

The DSI is the first instrument developed specifically for comprehensive assessment of dysmenorrheal symptoms. It depicts the effects of cyclic menstrual pain of different severity on patients’ everyday functioning, activities and quality of life and can be used for women of diverse age, race/ethnicity and other socio-epidemiological characteristics as well as for women with and without other gynecological conditions. DSI can be used at different phases of the menstrual cycle (during menses and the rest of the cycle) as two DSI versions (on-menses for daily measurements and off-menses when patients recall the most recent menstrual period) have been developed and evaluated using rigorous methods [16,17,26].

As the dysmenorrheal pain occurs only during menstruation and decreases as the menstruation passes the on-menses version of DSI is generally more reliable as it is less subject to recall bias. Still, the reliability of off-menses DSI version filled-in after the end of symptoms was also proven to be acceptable in all previous validation studies. Moreover, the fact that in previous validation studies no ceiling and floor effects were registered showed that DSI is sensitive to detect significant changes in symptoms intensity over time. Therefore, the on-menses version is better for direct assessment of symptoms in clinical settings while the off-menses version is applicable for investigating long term effects of dysmenorrhea on patients’ quality of life [16,17,26].

During the process of translation and cross-cultural adaptation for Serbian population no significant changes in items were made and all of the items were kept as integral parts of the scale. In other validation studies minimal changes of the items were performed, but some items (item #6) were deleted due to environmental and cultural differences. In some studies DSI items #7 and #8 (leisure and social activities) had low CVRs, but majority of patients marked these two items as necessary and consequently they were retained for comprehensiveness [16,26].

All of the previous validation studies of the DSI confirmed its single factor structure and adequate measurement properties. In the original validation study RMSEA ranged from 0.07 to 0.11 while all other fit indices were >0.9. The Brazilian validation study showed that DSI single factor structure explained 82.47% of variance with high values of investigated fit indices (>0.9) and RMSEA <0.06 which proved its good structural validity [17,26]. After appropriately setting the analysis parameters to suite our sample we also obtained excellent fit indices. High values of Cronbach’s alpha coefficient obtained both in the original and previous validation studies (>0.87) indicated adequate internal consistency while [17,26]. Previous validation studies showed that DSI is positively and strongly correlated with SPS-6 and with WHO-DAS [26]. In our study a significant association of DSI and DASS-21 was found. All of these findings confirm appropriate validity evidence based on internal structure of the DSI.

According to the available literature this is one of the few translation and complete validation studies of the DSI questionnaire. The main strength the research is strict validation procedure.

Still, our analysis has some limitations. The applied sampling procedure with strict inclusion and exclusion criteria might have caused sampling bias. The low threshold of symptoms frequency and intensity could have potentially diluted the results and a more stringent criterion might have been more appropriate to investigate symptoms interference. However, we specifically intended to include all women that have any symptoms of dysmenorrhea, even minimal and rare, as generally majority of dysmenorrhea patients do not have severe symptoms. Nevertheless, majority of investigated women reported having dysmenorrhea since the time of menarche during almost all cycles with intensive but bearable pain level, making the sample adequate for scale validation.

In addition this survey had a non-probabilistic sample that included only female health sciences students from a single University. We chose to examine health science students as medicine, dentistry and nursing represent the largest section at the University compared to all other faculties. Moreover, technical and natural sciences at the University are generally attended by male students due to social and cultural settings. It could be approximated that around 2000 female students are enrolled in health sciences in five Universities in Serbia overall. The ratio of students attending health sciences vs. other faculties as well as males vs. females is similar in all Universities in Serbia. Although our sample included a large number of students (350, i.e., 17.5% of all Serbian female health science students) from various socioeconomic backgrounds making the sample representative of healthy young Serbian women with similar age and education levels the representativeness of the obtained results is limited for the broader Serbian population of women with different ages, educational backgrounds not seeking higher education, or without any medical knowledge. Further studies on different populations are needed.

Moreover, the test‒retest reliability was not currently evaluated. We have performed the retest analysis but after the intervention for reducing dysmenorrhea symptoms. We are planning to report those findings in the upcoming study. Another study limitation is the fact that only 12% of investigated students had menstruation at the time of the survey while all others filled-in the off-menses DSI version. However, our study aimed to validate the Serbian DSI for the general healthy young female population and not to investigate symptoms per patient. Consequently it was performed among women who had dysmenorrhea mostly for years and therefore we considered that the recall bias was minimal. This methodology was also used in other similar studies [27].

## Conclusions

The Serbian version of the Dysmenorrhea Symptoms Interference Scale was proven to be a valid and reliable specific instrument for assessing the impact of menstrual pain on a woman’s quality of life. As DSI has adequate psychometric properties it can be applied both in research and in broad clinical use to assess and classify dysmenorrhea symptoms of women from Serbia as well as to develop and test interventions for dysmenorrhea.
